# Complex bile duct network formation within liver decellularized extracellular matrix hydrogels

**DOI:** 10.1038/s41598-018-30433-6

**Published:** 2018-08-15

**Authors:** Phillip L. Lewis, Jimmy Su, Ming Yan, Fanyin Meng, Shannon S. Glaser, Gianfranco D. Alpini, Richard M. Green, Beatriz Sosa-Pineda, Ramille N. Shah

**Affiliations:** 1 Biomedical Engineering, Northwestern University, Evanston, IL, USA; 2 Simpson Querrey Institute, Northwestern University, Chicago, IL, USA; 3 Research Central Texas Veterans Health Care System, Temple, TX, USA; 4 Baylor Scott & White Health Digestive Disease Research Center, Temple, TX, USA; 5 Medical Physiology, Texas A&M University College of Medicine, Temple, TX, USA; 6 Division of Gastroenterology and Hepatology, Northwestern University, Chicago, IL, USA; 7 Nephrology, Northwestern University, Chicago, IL, USA; 8 Materials Science and Engineering, Northwestern University, Evanston, IL, USA; 9 Surgery (Transplant Division), Northwestern University, Chicago, IL, USA

**Keywords:** Tissues, Tissue engineering

## Abstract

The biliary tree is an essential component of transplantable human liver tissue. Despite recent advances in liver tissue engineering, attempts at re-creating the intrahepatic biliary tree have not progressed significantly. The finer branches of the biliary tree are structurally and functionally complex and heterogeneous and require harnessing innate developmental processes for their regrowth. Here we demonstrate the ability of decellularized liver extracellular matrix (dECM) hydrogels to induce the *in vitro* formation of complex biliary networks using encapsulated immortalized mouse small biliary epithelial cells (cholangiocytes). This phenomenon is not observed using immortalized mouse large cholangiocytes, or with purified collagen 1 gels or Matrigel. We also show phenotypic stability via immunostaining for specific cholangiocyte markers. Moreover, tight junction formation and maturation was observed to occur between cholangiocytes, exhibiting polarization and transporter activity. To better define the mechanism of duct formation, we utilized three fluorescently labeled, but otherwise identical populations of cholangiocytes. The cells, in a proximity dependent manner, either branch out clonally, radiating from a single nucleation point, or assemble into multi-colored structures arising from separate populations. These findings present liver dECM as a promising biomaterial for intrahepatic bile duct tissue engineering and as a tool to study duct remodeling *in vitro*.

## Introduction

Significant progress has been made over the past several decades in the field of liver tissue engineering and regenerative medicine. However, the majority of current approaches focus on manipulating hepatocytes in ectopic sites^1–3^. Such approaches have the potential to treat metabolic diseases and protein synthesis deficiencies (e.g. hemophilia), provided that the biliary tree is physiologically intact. However, patients suffering from end stage liver disease (especially cholangiopathies) require orthotopic liver transplantation or living donor liver transplantation for restoration of hepatobiliary function. Liver tissue engineering strategies aimed at producing transplantable tissue must therefore restore the entire liver architecture, including the biliary tree. The intrahepatic biliary tree is responsible for concentrating and transporting bile from the blood stream, while extrahepatic portions store and secretes bile into the duodenum^4^. Developmentally, the biliary tree arises from the ventral foregut endoderm with hepatocytes and intrahepatic cholangiocytes having separate lineages from extrahepatic cholangiocytes^5^. The mature intrahepatic biliary epithelium displays a phenotypic heterogeneity, with small cholangiocytes populating the periphery of the biliary tree with limited capacity for secreting bile and little to no response to hormones like secretin or somatostatin^6,7^. In contrast, large cholangiocytes populate larger diameter intrahepatic and extrahepatic ducts and display a mature cholangiocyte phenotype by being active in bile transport. Morphologically, small and large cholangiocytes display obvious size differences^8^. In addition, the functional differences between small and large intrahepatic cholangiocytes play large roles in normal liver physiology and in response to liver injury^9^. Small cholangiocytes normally serve to line the bile duct tube so that bile may be modified by large cholangiocytes once it flows into larger diameter ducts. Small cholangiocytes are also normally mitotically inactive, but may proliferate and differentiate into large cholangiocytes as a response to liver injury^10^. The heterogeneity of the biliary tree has yet to be addressed from a tissue engineering perspective or in *in vitro* models of development or remodeling.

Strategies to re-create the extrahepatic and intrahepatic biliary tree have been met with varying levels of success^11,12^. Sampaziotis *et al*. successfully reconstructed mouse gallbladder and common bile duct using an organoid approach^13–17^. Organoid approaches have led to numerous advances in biliary tree regenerative medicine, with several approaches capable of producing heterogeneous cellular structures^18,19^. Engineering the intrahepatic biliary tree is particularly challenging due to both its size and complexity: the human intrahepatic bile ducts range in size from millimeters to less than 15 micrometers in diameter^20^. The biliary tree permeates every level of liver anatomy from the hepatic hilum to the liver lobule. Using the relatively popular top-down approach of whole organ decellularization and recellularization, the liver vasculature has been successfully repopulated with endothelial cells^21–24^, however, recellularization using a similar approach for the biliary tree has been met with limited success^25^. This may be due to several factors ranging from the fastidious nature of cholangiocytes to the tortuous and complex architecture of the biliary tree.

**Figure 1:**
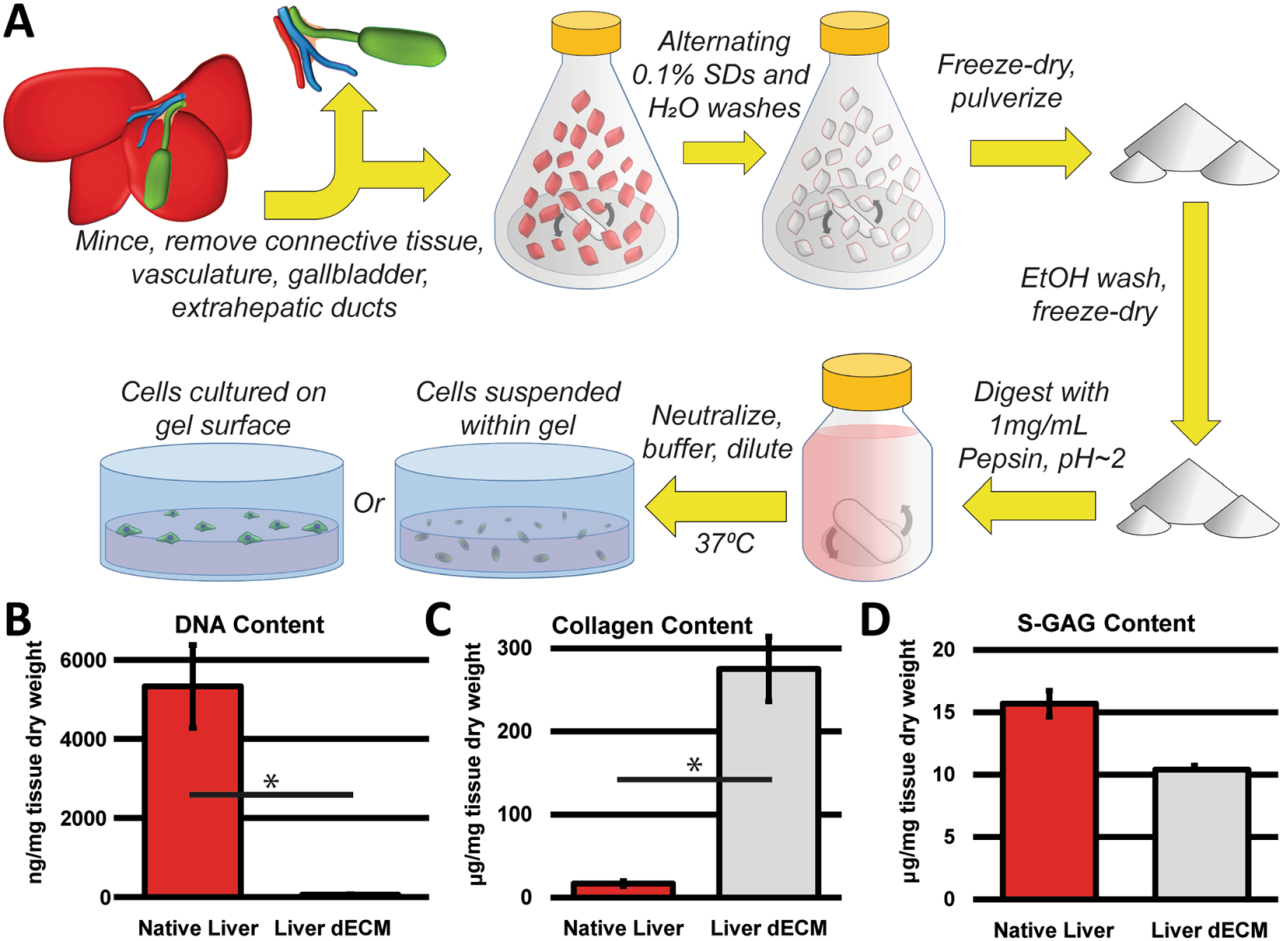
Liver Decellularization and Cell Encapsulation. (**A**) Schematic of the liver decellularization and cell encapsulation process. Porcine liver is subjected to mincing and alternating washes of SDS and water, followed by lyophilization and milling, and sterilization. Liver dECM is digested for 48 hours at room temperature under heavy agitation. Cholangiocytes in media are either combined with the neutralized, cold pre-gel for encapsulation, or plated on top of a pre-formed gel. (**B**) DNA content of liver dECM is ~1% that of native liver, indicating complete decellularization. (**C**) Collagen content by weight is increased in liver dECM after decellularization due to removal of cellular material. (**D**) ECM-based sulfated glycosaminoglycans (GAGs) are retained after decellularization. Error bars ± SD, n = 3, *p < 0.05.

Bottom-up approaches to intrahepatic biliary tree tissue engineering have sought to employ hydrogel matrices. The extracellular matrix (ECM) of the developing liver plays an integral role in the formation and maturation of the biliary tree^26,27^. The biliary tree forms from a bilayer of hepatoblasts surrounding the portal vein, termed the ductal plate, which become polarized and surrounded by a basal membrane through interactions with the portal mesenchyme. This process is regulated by Jagged/Notch signaling, TGFβ signaling, and WNT/β-catenin and involves interactions with the surrounding ECM^28^. ECM mimics such as type 1 collagen or Matrigel in either culture of cholangiocytes or in co-culture with hepatocytes have shown promising results for intrahepatic biliary tree tissue engineering^11,29^. Primary cholangiocytes are known to possess a degree of *in vitro* morphogenic capability, however the degree of duct formation seen in Matrigel or type 1 collagen is rudimentary^29–33^. Tissue-specific liver decellularized ECM (dECM) hydrogels have not yet been investigated for intrahepatic bile duct tissue engineering research. Advantages of liver dECM include its lack of immunogenic cellular material such as DNA that would preclude xenogeneic transplantation (Fig. 1B) along with preservation of several biochemical and mechanical characteristics beneficial to both biliary tree tissue engineering and the study of its remodeling^34–38^. ECM processed in this manner is composed primarily of collagen, which is suitable for long-term cultures (>1 month) and exhibits well-studied solubilization and gelation properties facilitating its use in a number of tissue engineering approaches^34,37,39^. In the present study, we explore the use of liver dECM hydrogels as an inductive environment for *in vitro* bile duct formation. Our results not only show promise for future applications in intrahepatic bile duct tissue engineering, but also in the study of bile duct remodeling and cholangiocyte-matrix interactions.

## Results

### Liver decellularized extracellular matrix (dECM) processing

A hydrogel derived from porcine liver dECM was fabricated using standard detergent washing and enzymatic digestion methods (Fig. 1A)^40,41^. Because of the emphasis of structural and insoluble matrix signaling components in bile duct development, the method developed by Freytes *et al*. was chosen as opposed to other methods that may retain a higher proportion of soluble ECM components^42^. Due to concerns over toxicity of residual sodium dodecyl sulfate (SDS), we appended standard methods with a final wash of the lyophilized dECM powder in 70% ethanol, which serves the dual purpose of SDS removal and terminal sterilization (Supplementary Fig. 1a)^43^.

Pepsin digestion and dECM solubilization at acidic pH is often reported to be inefficient after 48 hours of digestion at room temperature, necessitating centrifugation to remove undigested (or under-digested) particles. These undigested pieces likely consist of dense structural proteins, the removal of which would lead to an incomplete representation of liver ECM in the resulting gel. We therefore optimized our digestion protocol by reducing digestion volume and maximizing agitation, without the introduction of bubbles. The resulting digest is a viscous solution free of undigested particulate. Upon neutralization and cell encapsulation, the gelation reaction at 37 °C stabilizes in under 10 minutes, forming a randomly-oriented fibrous network of matrix proteins that is robust enough to prevent any cell settling (Supplementary Fig. 1b,c). Because liver tissue itself is primarily composed of cellular material, quantification of collagen by biochemical measurement of hydroxyproline content indicates a relative increase of the overall weight percentages after decellularization (Fig. 1C). However, sulfated glycosaminoglycan (S-GAG) content by weight is reduced, as some cellular material, such as glycoproteins, contain GAGs (Fig. 1D).

### Cholangiocyte encapsulation and duct formation

**Figure 2:**
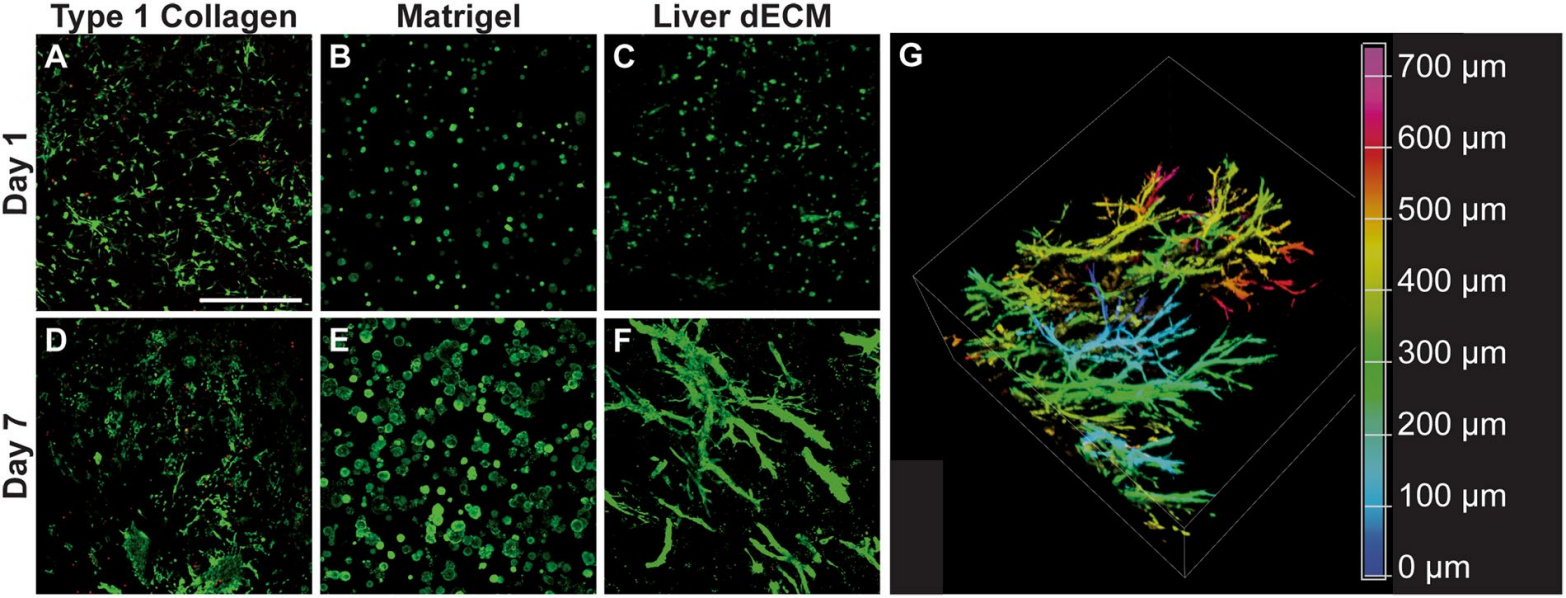
Cholangiocytes Encapsulated in Liver dECM Spontaneously form Duct-Like Structures. (**A**–**C**) Cholangiocytes encapsulated within type 1 collagen, Matrigel, or liver dECM are relatively similar after 1 day in culture. (**D**–**F**) After 7 days, cholangiocytes in collagen will proliferate and spread, while Matrigel cultures will form cysts, in contrast to liver dECM structures which form complex branching structures. (**G**) Duct structures will branch into complex 3-dimensional networks within thick gels over longer time periods (21 days). Color corresponds to z-depth into a confocal Z image stack. All samples are confocal z-stack maximum intensity projections, visualized with live/dead (green/red) viability stain, scale bar = 500 μm.

Immortalized murine small cholangiocytes (SV40SM^44^) were encapsulated within liver dECM gels and compared to those encapsulated in either Matrigel or purified type 1 collagen gels. Cholangiocytes within the liver dECM gels showed high viability and assembled into complex branching duct-like structures on scales not previously observed. This phenomenon was not observed in either the Matrigel or purified collagen gels (Fig. 2A–F). Type 1 collagen is the primary structural ECM protein in most tissues including the liver. The lack of any organized structure of the SV40SM cholangiocytes within the type 1 collagen gels indicate that additional matrix proteins and soluble factors are necessary for bile duct formation, and SV40SM cells appear to lack the capacity to sufficiently produce such proteins under these culture conditions. Matrigel is a commercially available matrix that closely approximates the components of the basement membrane. SV40SM cells show a stereotypical cyst-like structure formation in Matrigel cultures (Fig. 2E); however, branched structures are not observed. The biliary tree itself is lined with a basement membrane that aids in isolating the bile duct lumen from the bloodstream, and with these basement membrane components in Matrigel (collagen IV, laminin, etc.) are necessary for cholangiocyte polarization and function^30,45^. Immunostaining of SV40SM encapsulated within liver dECM for laminin indicates a diffuse background staining (Supplementary Fig. 2), with concentrations around the exterior of the hollow (cyst or duct cross sections) structures.

We hypothesize that the combination of matrix components within liver dECM is unique in its ability to cause the observed assembly of cholangiocytes into bile duct structures. Many different dECM hydrogels have been shown to retain matrix bound and soluble tissue-specific signaling components, however the presence of residual cellular proteins has not seen the same amount of attention^46,47^. Notch signaling is known to be critical to biliary development and duct tube morphogenesis^48^. However, notch ligand (and notch receptor) proteins are both located within the plasma membrane, which is theoretically completely removed after completion of the decellularization process. However, we observe slowed duct formation after incubation with the ɣ-secretase inhibitor L-685, 458 (Supplemental Fig. 3a). While we see a reduction in the number of duct structures formed (Supplemental Fig. 3b), we do not observe a reduction in the number of branches per structure (Supplemental Fig. 3c). Regardless, notch signaling appears to be taking place in these cultures. However, assays for the notch ligand Jagged1 indicate jagged is not detectible within dECM alone but is detectible in both cultures of SV40SM and SV40LG (Supplemental Fig. 3d). Because cholangiocytes do not normally express notch ligands, Jagged1 presence may be an indication that SV40SM cells are heterogeneous, containing a mesenchymal component, or there is an epithelial to mesenchymal transition (EMT) taking place in some or all SV40SM. Additionally, we observe a significant decrease in Notch1 protein production after 1 week in dECM encapsulation compared to 2D cultures (Supplemental Fig. 3e). Interestingly, Notch1 was detectible in dECM alone. This may indicate that Jagged1 (and other notch ligands) are present but not detectible due to the porcine origin of the dECM and the murine reactivity of the assay. More in-depth investigations into residual cellular proteins within dECM are necessary.

**Figure 3:**
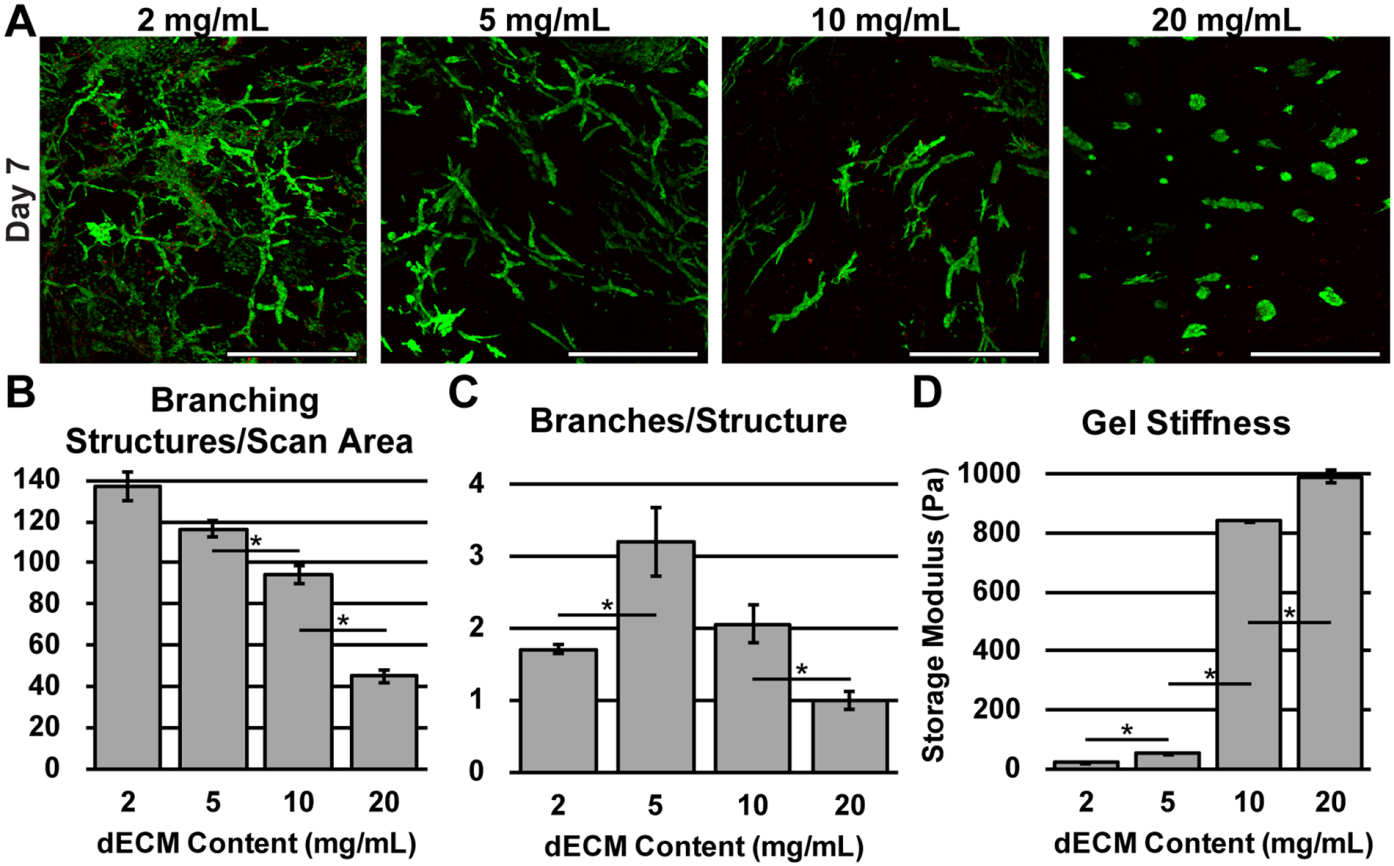
Liver dECM Content Influences Duct Formation. (**A**) Varying proportions of dECM influence formation of duct structures. All samples are confocal z-stack maximum intensity projections, visualized with live/dead (green/red) viability stain. (**B**) Quantified number of branching structures per image, indicating a reduction with increasing dECM content. (**C**) Quantified number of branches per structure. (**D**) Storage modulus (solid-like properties) of dECM hydrogels of different mass fractions. Error bars = ± SD, n = 3. Scale bar = 500 μm. *p < 0.05.

While some researchers are able to observe *in vitro* morphogenesis using primary isolated epithelial tissue^49^, or in the presence of feeder-cell co-culture^50^, our study demonstrates cholangiocytes branch from single cell suspensions as opposed to primary isolated bile duct tissue^51^. Cultures grown for extended periods of time (21 days) display variations in branch thickness (Supplementary Fig. 4) and increasing gel volume facilitates branching in three dimensions (3D) (Fig. 2G) as opposed to across the interior of a thin gel (Supplementary Fig. 4d). When comparing the behavior of SV40SM cells on the surface of a 3D dECM gel as opposed to encapsulation, culturing on the surface led to budding into the gel (Supplementary Fig. 5) and short branch formation. To test what effect dECM weight percentages have on duct formation, we encapsulated SV40SM in varying concentrations of dECM. After 7 days, duct formation is severely stifled in higher concentrations, as only cellular aggregates are visible (Fig. 3A). Quantification of number of branches per image scan area indicate an inverse relationship between dECM weight percent and the number of branches formed (Fig. 3B). However, reducing dECM weight percent would eventually lead to a gel too weak to sustain long term culture. The number of branches within a single structure reaches a maximum at 5 mg/mL dECM (Fig. 3C). The reduced number of branches in lower concentrations is possibly due to the increased number of structures within the gel competing for space. The reduction in number of ducts and branches in higher concentrations is likely caused by the increase in ECM that must be degraded to allow for infiltration and migration of cholangiocytes as well as increased dECM stiffness. Substrate stiffness is known to have significant effects on cellular behavior^52^, and the increase in shear modulus observed when increasing dECM concentration shown in Fig. 3D is likely also responsible for the stifled duct formation.

### Cholangiocyte phenotypes and ability to form bile ducts

**Figure 4:**
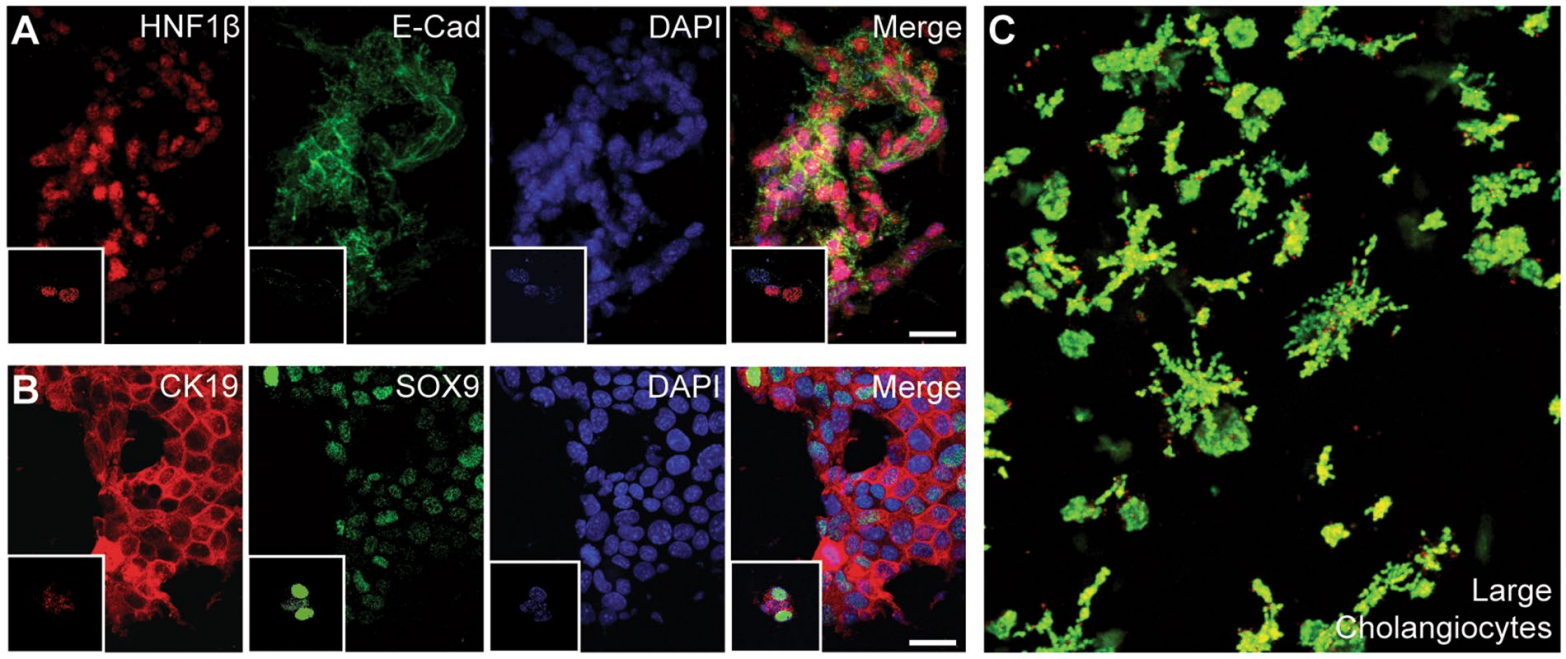
Cholangiocyte Phenotype is Essential for Duct Formation. (**A**) Duct-like structures formed by small cholangiocytes express HNF1β, indicative of intrahepatic cholangiocytes after 7 days in culture. (**B**) Cells also express SOX9 and CK19, definitive indication that the cholangiocyte phenotype is maintained after 7 days. Insets in (**A**) and (**B**) show staining after 1 day in culture. (**C**) Confocal z-stack maximum intensity projection of live/dead stained large cholangiocytes (SV40LG) encapsulated within liver dECM, ducts do not form after 7 days. Scale bars = 25 μm.

Small cholangiocytes are a subset of cells that populate the biliary tree, and display a distinct phenotype from large cholangiocytes^7,44,53^. Large cholangiocytes function by responding to the gastrointestinal hormone secretin and by modifying bile concentration through expression of CFTR and Cl^−^/HCO_3_^−^ exchanger and aquaporins, properties that small cholangiocytes lack^54^. Small cholangiocytes, however, are hypothesized to retain the capacity to repopulate an injured biliary tree or potentially the entire liver^6,55–57^. Small cholangiocytes can be assigned a committed progenitor phenotype, whereas large cholangiocytes represent an attractive cell source (via cholecystectomy or biopsy) for tissue engineering^17^. We therefore sought to investigate the potential of an immortalized murine intrahepatic large cholangiocyte cell line (SV40LG) to form duct-like structures in comparison to small cholangiocytes. We observed that within the liver dECM SV40LG do not form the extensive branching network seen with the small cholangiocytes, and instead, are observed to proliferate and migrate without any semblance of organization (Fig. 4C). Because both small and large cholangiocytes will display different proliferative responses to injury *in vivo*^53^, proliferation (via DNA content) of both SV40SM and SV40LG was measured in standard 2D and dECM encapsulation conditions (Supplementary Fig. 6). SV40 small and large cholangiocytes proliferate at the same rate when encapsulated within liver dECM, indicating that the formation of ducts from SV40SM is not due to a difference in proliferative capacity from SV40LG.

**Figure 5:**
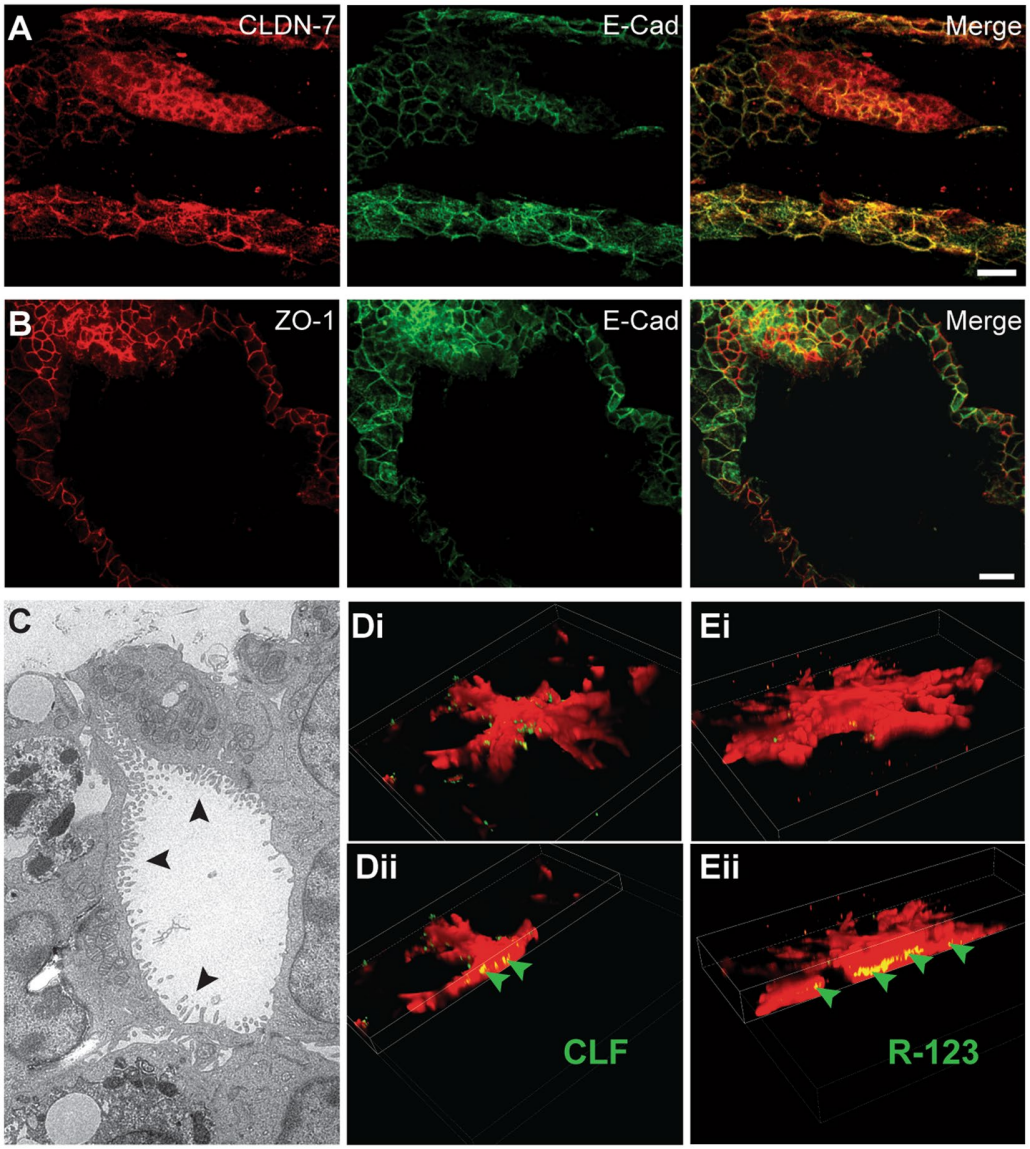
Duct-Like Structures Show Functional Maturation. (**A,B**) E-Cadherin, Claudin-7, and Zonula Occludens-1 (ZO1), are all expressed in duct-like structures, indicating maturation of tight junctions. (**C**) Apical microvilli (arrows) are visible under TEM in cross-sections of duct/cyst lumen. (**D,E**) mCherry-expressing SV40SM structures incubated in either CLF (Di) or R123 (Ei). Cutaways (Dii, Eii) indicate apical secretion (arrowheads) of the dye into an interior lumen, which appears yellow due to red/green overlap. Scale bars = 25 μm.

The ability of SV40SM to form complex branching structures within dECM hydrogels is evidence of maintenance of small cholangiocytes’ plasticity in an SV40 immortalized state. However, the production of Jagged proteins is indicative of a possible EMT. Therefore, to evaluate whether SV40 immortalization causes any deviation from the cholangiocyte phenotype, we performed several immunostaining experiments. SOX9 is expressed in cholangiocytes and in subsets of periportal hepatocytes during development or injury^28^. However, when SOX9 is expressed concurrently with CK19, it is indicative of a cholangiocyte phenotype (Fig. 4A). Additionally, we observed expression of HNF1β concurrently with high expression of E-Cadherin (Fig. 4B). HNF1β is another marker for hepatocytes and intrahepatic cholangiocytes, from which these cells are derived^58^, while E-cadherin is indicative of adherens junction formation commonly seen in epithelial cells. Additionally, we show maturation of tight junctions with co-localization of E-cadherin and claudin-7 (Fig. 5A). Claudin-7 is unique to cholangiocyte tight junctions within liver tissue, indicating SV40SM are not de-differentiating to a hepatoblast or trans-differentiating to a hepatocyte^59^. Additionally, E-cadherin co-localizes with ZO1, indicating further tight junction maturation (Fig. 5B). Formation, maturation, and maintenance of tight junctions is a critical aspect of duct function.

Polarization of cholangiocytes is also essential to modification and secretion of bile. In both Matrigel and dECM cultures, we observed formation of apical microvilli in cyst and duct cross-sections evident via transmission electron microscopy (TEM), respectively (Supplementary Fig. 7, Fig. 5C). Small cholangiocytes have the ability to polarize, however, their cAMP-dependent secretory capacity is limited^60^. To evaluate secretion, stably transfected mCherry expressing SV40SM were incubated with cholyl-lysyl-fluorescein (CLF) or Rhodamine-123 (R123). CLF uptake into the cell occurs via organic anion transporter proteins (OATPs) and apical secretion is via multidrug resistance protein 2 (MRP2), while secretion of R123 occurs via multidrug resistance protein-1^61,62^. After incubating with CLF or R123, cells were cultured under normal growth conditions to allow for apical secretion. Apical secretion of CLF or R123 into the interior of a lumen of mCherry-SV40SM is apparent by localization of the green fluorescent dye within the red duct-like structures, which appear yellow due to overlap (Fig. 5D,E, arrowheads). Of note, however, is that CLF and R123 taken up by mCherry-SV40SM do not show a continuous lumen, which would be a single green fluorescent tube within a red duct-like structure. Instead we observe discrete pockets of CLF and R123 localization within mCherry-SV40SM structures. The lumen discontinuity is potentially reflective of the immature (i.e. small) state of these ducts and is a property of early duct remodeling *in vivo*.

### Morphology and assembly of *In vitro* formed bile ducts

**Figure 6:**
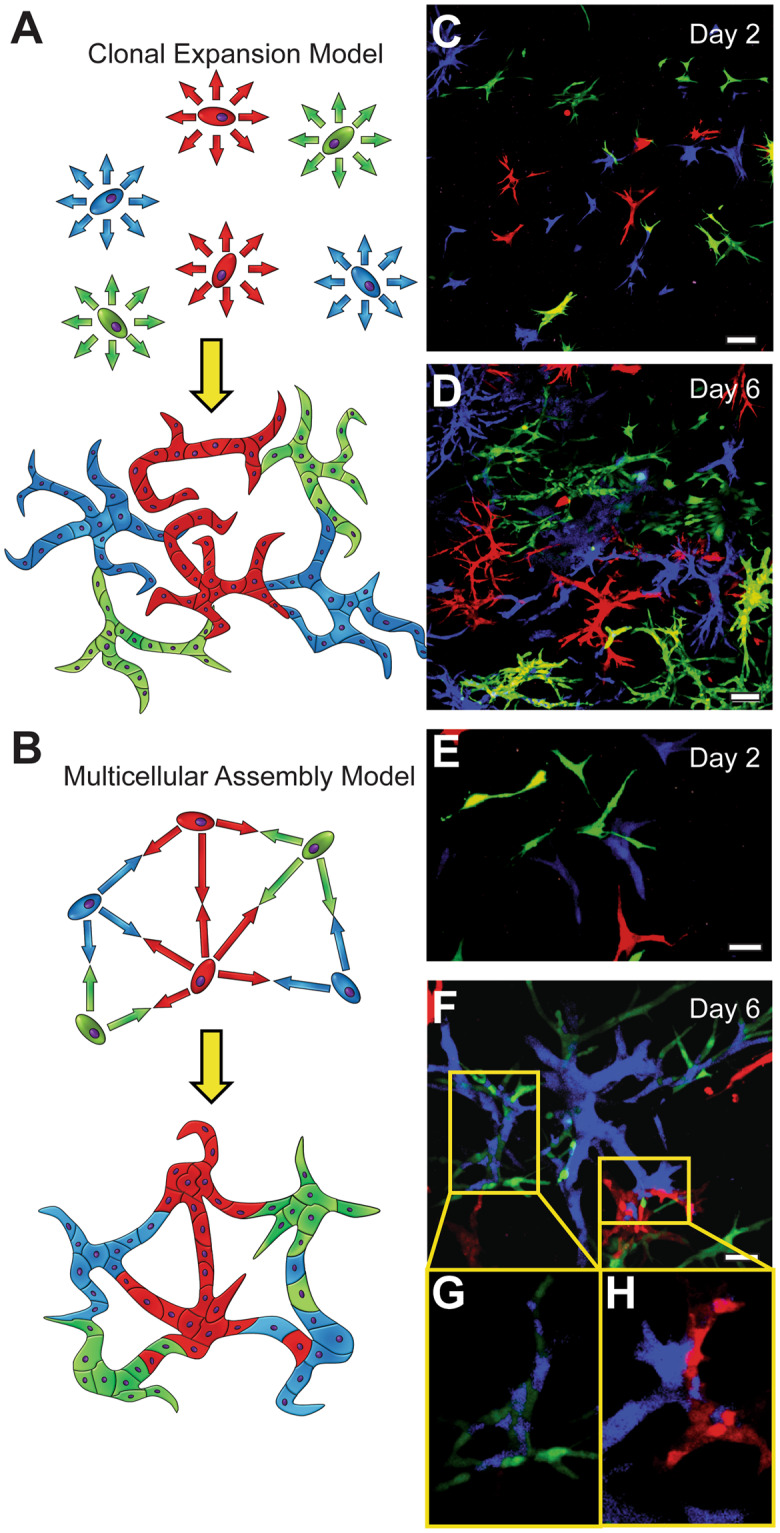
Ducts Branch Clonally, Assemble to Form Networks, or a Combination of Both. (**A**) Schematic of one proposed mechanism of duct formation wherein single cells give rise to clonal populations of branching structures composed of a single color. (**B**) Alternative proposed mechanism in which multiple cells assemble to form ducts with mixed lineages wherein resulting structures display a confetti or mosaic appearance. (**C,D**) Low magnification maximum intensity Z projection image of Identical populations of cholangiocytes expressing 1 of 3 different colors (red/green/blue, mCherry/eGFP/Azurite) mixed within liver dECM gel after 2 (**C**) and 6 (**D**) days in culture. Structures superficially resemble model **A**. Higher magnification image at 2 (**E**) and 6 (**F**) days indicate mixing and multicellular assemble (model **B**). Single Z slices (**G,H**) indicate the mosaic appearance is not an artifact of maximum intensity z projection. Scale bar **C,D** = 100 μm, **E,F** = 50 μm.

**Figure 7:**
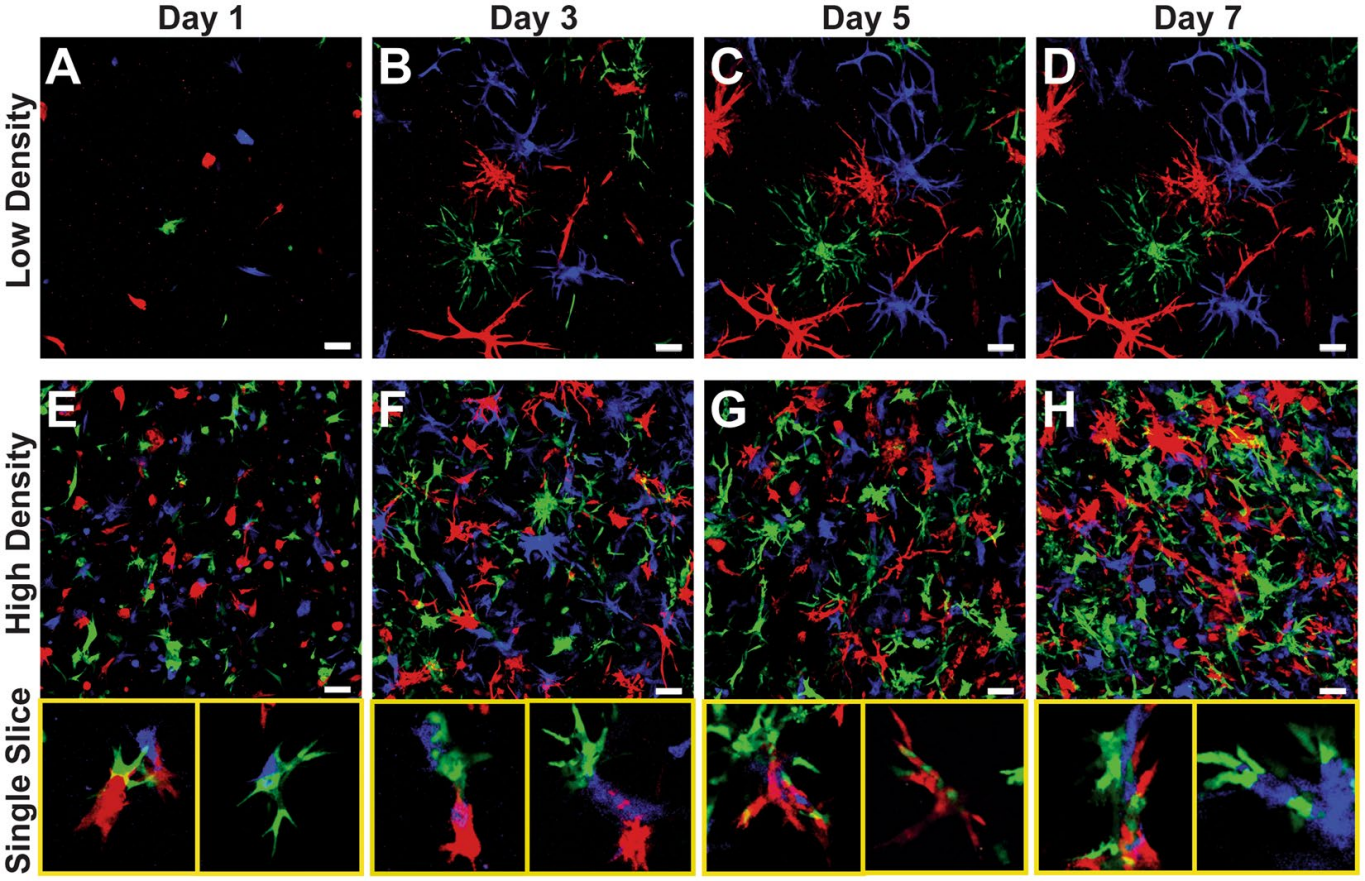
Duct Assembly Model Depends on Proximity. Multipoint imaging of low cell density (5e4 cells/mL) cultures (**A–D**) and high cell density (1.5e6 cells/mL) cultures (**E–H**). Low density cultures show clonal branching from single cells suspended within the gel, while multi-colored structures are apparent in high density cultures after only a single day in culture because of their close proximity. Representative single z-slices of high density cultures indicate that structures composed of two or three colors are fairly common at all time points. Scale bars = 100 μm.

We sought to further understand the duct forming phenomenon we observed in dECM cultures by placing it in a broader context within liver development and remodeling. Development of the intrahepatic biliary tree proceeds *in utero* from a bipotent progenitor along the portal tract^27^. Single histological sections of the ductal plate indicate hollow tubule formation, however what is unclear is how the entire biliary tree achieves lumen continuity during development or remodeling. We hypothesize this is by either longitudinal formation beginning at the liver hilum, or the assembly of multiple tubules within the developing liver. The duct formation process observed within liver dECM cultures can serve as a model for remodeling, which we initially anticipated to follow one of two schemes: either single cells suspended within dECM gels expand clonally in a ‘tree-like’ manner (Fig. 6A), or multiple cells assemble together to form networks (Fig. 6B), similar to endothelial cells cultured on the surface of Matrigel^63^. To test these hypotheses, we developed SV40SM cells stably transfected with either red (mCherry), green (eGFP), or blue (Azurite) fluorescent proteins and encapsulated them in equal proportions in liver dECM. Resulting structures would either be composed of a single color (‘tree-like’ clonal expansion) or would display a multi-colored confetti or mosaic-like appearance (‘multicellular assembly’). Single color experiments indicate that all fluorescent cell lines retain the ability to form duct-like structures (Supplementary Fig. 6). Initial multipoint imaging of multi-color cultures supports the clonal expansion model, evident by many branching structures composed of only one color (Fig. 6C,D). The *in vivo* correlate to this would be longitudinal duct formation from the hilum. A separate scan area of the same experiment however reveals duct structures composed of multiple colors (Fig. 6E,F), providing support for a network model. Cells depicted in Fig. 5E begin culture adjacent to one another and result in a confetti-like appearance after prolonged culture (Fig. 6F). To demonstrate that these images are not artifacts of Z stack compression, single Z-slices at different heights were isolated, and indicate multi-colored structures of green and blue in Fig. 5G, or of red and blue in Fig. 5H. Supplementary Video 1 shows a slice-by-slice scroll of the entire structure and a 3D rotation. Additionally, several multi-colored ducts were observed to merge with one another over long culture periods (Supplementary Fig. 7), but this does not appear in 100% of cases. The proximity of cells during duct formation seems to govern their assembly process. In order to test this hypothesis, we repeated this experiment using either a high density of cells (1.5e6 cells/mL gel) or a low density of cells (5e4 cells/mL gel) under the assumption that a higher density would increase the likelihood of cholangiocytes being forced into close proximity to one another, while cells in isolation will branch out clonally. Figure 7 summarizes these results and indicates that multi-colored structures are apparent and numerous after even a single day in high density culture, while only a single multi-colored duct was observed in low density cultures (not pictured). Single z-slices (Fig. 7 bottom panels) of high density cultures show examples of multi-colored structures at corresponding time points.

## Discussion

In this study, we demonstrated that liver dECM hydrogels impart a unique morphogenic and functional effect on encapsulated cholangiocytes. Liver dECM hydrogels were produced using standard detergent washing and pepsin digestion approaches. We found that SV40 immortalized small cholangiocytes require the specific components of liver dECM to branch, and purified collagen 1 or Matrigel are incapable of leading to the same response. Ducts arising from SV40SM cells show phenotypic stability in addition to polarization and transport capability. Furthermore, we are able to track duct formation *in vitro* to study how multiple cells assemble and multiply to form complex branching structures. The approaches developed here are some of the first steps into the development of engineered liver tissue capable of transporting bile.

Notably, we demonstrate duct formation without extensive modification of culture media. Recent advances in organoid and stem cell research have sprung from empirically designed and fairly complex media supplements to optimize results. Our usage of a comparatively simple media cocktail indicates that the liver dECM structural components and residual soluble factors are sufficient to lead to complex duct formation in SV40SM cells. While other approaches have been able to demonstrate branching from human-derived iPS cells, these approaches required Matrigel in combination xenogeneic co-culture of mouse cell lines, adding an additional layer of complexity and precluding future experiments into tissue engineering applications^50^. Decellularized ECM has been employed clinically for several years due to its lessened immunogenicity and enhanced bioactivity^47^. Furthermore, the data presented here also reiterates the differences between small and large cholangiocytes. Previous research has demonstrated differences in susceptibility to injury and proliferation of small and large cholangiocytes^10,53,64^. Small cholangiocytes, modeled in these experiments by SV40SM, normally do not proliferate *in vivo*. However, small cholangiocytes can proliferate and respond to hormone stimulation after loss of large cholangiocytes, effectively repopulating an injured biliary tree. While SV40SM and SV40LG proliferate identically within dECM culture, dECM encapsulation may be mimicking an injured environment, causing SV40SM to form branching structures in a process analogous to early stages of remodeling post-injury. Transport of fluorescent dyes CLF and R123 indicate an additional level of function normally reserved for large cholangiocytes, signifying possible small-to-large differentiation. Future studies into small and large co-culture can potentially demonstrate how these two cell types may interact in the dECM hydrogel environment, and if the presence of large cholangiocytes prevents duct-formation seen with small cholangiocytes. Liver dECM hydrogels can therefore serve as a powerful tool to study the crucial role the ECM plays in liver remodeling and the interplay between small and large cholangiocytes.

Our experiments with varying weight percentages of dECM show that dECM conditions can influence and suppress duct formation. This can be further applied to model disease progression *in vitro* with other experiments incorporating dECM isolated from diseased livers, or dECM adulterated with additional matrix components to simulate fibrosis like surplus collagen 1, for example. Future studies will additionally require investigation into what specific components within liver dECM lead to the branching phenomenon. Specifically, soluble TGFβ and Activin factors are known participants in duct development, while residual VEGF or HGF may participate in enhanced proliferation and migration ability of SV40SM cells^27,65,66^. The ability of soluble factors like VEGF or HGF to bind to ECM proteins is well studied, and their presence in dECM has been previously demonstrated^46,47^. Notch signaling also is known to be critical to duct development^48^, but notch ligands (and receptors) are cell surface proteins theoretically removed during the decellularization process unlike matrix-bound growth factors. Residual Notch1 receptor presence within dECM indicate that leftover cellular proteins, in addition to ECM, ECM-bound proteins, and even extracellular vesicles may all be components of dECM contributing to its unique bioactivity. In addition to the presence of collagen, S-GAGs, and laminin, matrix components such as nidogen, elastin, and hyaluronan as well as specific collagen or laminin types should also be investigated. Proteomics strategies can help define important soluble components of dECM, however limitations arise when analyzing the insoluble component, which is thought to be critical for duct development. Targeted antibody-based approaches such as ELISAs or Western Blotting can be used in conjunction with specific developmental pathway inhibitors to definitively determine dECM components responsible for our observations. Additionally, subsequent approaches will supplement dECM with soluble factors to enhance duct maturation and functionality with the end goal of reaching a phenotypic heterogeneity mimetic of what is observed *in vivo*. The results demonstrated here show that small, but not large cholangiocytes, can lead to formation of complex networks of duct-like structures *in vitro*. Because large intrahepatic cholangiocytes are phenotypically similar to extrahepatic duct or gallbladder-derived cholangiocytes, these results indicate that extrahepatic cholangiocytes may not be the ideal cell type for intrahepatic bile duct tissue engineering.

Future experiments involving dECM gel culture will incorporate primary isolated small and large cholangiocytes. The SV40 immortalization is thought to be responsible for the enhanced proliferation, however the small cholangiocyte phenotype is ultimately what leads to the branching, as branching is not observed in large cholangiocytes. Experiments into primary culture will need to induce proliferation without SV40 immortalization to determine the potential of these cells for tissue engineering applications. Furthermore, multiple cell types should be incorporated from both tissue engineering and basic science standpoints. The developing biliary tree leads to the formation of the hepatic artery along the portal tract^26,27^. Co-culture of cholangiocytes with endothelial cells will enhance vascularization essential for tissue integration upon implantation, while also facilitating the study of coordinated duct/artery formation. Because both hepatocytes and intrahepatic cholangiocytes arise from bipotent hepatoblasts, it is unknown if co-culture can lead to the interface between cholangiocytes and hepatocytes, known as “the Canals of Hering.” Hepatoblast dECM culture has the potential to lead to heterogeneous structures composed of both hepatocytes and cholangiocytes answering many questions relevant to lobule development, such as whether a phenotypic intermediate is necessary for the formation of the Canals of Hering. These initial investigations into the use of liver-specific dECM for *in vitro* duct formation further demonstrate the potency of utilizing tissue-specific dECM for tissue engineering and also its potential use for expanding our understanding of developmental/remodeling processes that occur during embryogenesis or upon organ injury.

## Materials and Methods

### Liver Decellularization, Digestion, and Analysis

Livers were harvested immediately post-euthanizing from 3-4-month-old female Yorkshire pigs due to be sacrificed as a part of the Feinberg School of Medicine’s Surgical Simulation Lab. All experiments were approved by and performed within the guidelines and regulations of the Northwestern University IACUC Tissue Sharing Program for USDA covered animals. Livers were frozen at −80 °C until decellularization. Decellularization proceeded using a modified detergent and water washing protocol^40,67^. Briefly, tissue was sliced into ~1 mm cubes and subjected to repeated washes in deionized water to remove blood and induce hypotonic cell lysis. Tissue was then subjected to a 0.1% SDS (Sigma) wash overnight, followed by another series of water washes. The SDS/dH_2_O sequence was repeated once more prior to lyophilization and milling. Milled liver dECM powder was then subjected to a 70% ethanol wash serving dual purposes of sterilization and residual SDS removal^43^. The following lyophilization and digestion steps took place under aseptic conditions to maintain sterility. The sterilized powder was digested at a concentration of 10 mg/mL dECM and 1 mg/mL pepsin (Sigma) at 0.01 M HCl (Sigma) for 48 hours at room temperature under heavy agitation via magnetic stir bar. Pepsin digest was either frozen at −80 °C or immediately used for cell encapsulation. DNA content was assayed using PicoGreen dsDNA Assay Kit (ThermoFisher) as previously described for both native and decellularized liver samples (n = 3)^68^. Briefly, samples were digested overnight with Proteinase K (Sigma) and then subjected to the assay. Glycosaminoglycan (GAG) content of dECM was determined using dimethyl methylene blue assay as previously described (n = 3)^69^. Collagen content was quantified using Sircol collagen quantification assay (Bicolor) according to manufacturer specifications (n = 3). Residual SDS content was quantified using an SDS detection and estimation kit (G-Biosciences) according to manufacturer specifications (n = 3). DNA, Collagen, GAG, and SDS assays were analyzed using a Cytation 3 Plate Reader (BioTek). For SEM imaging, gel samples were fixed for 20 minutes in 2% glutaraldehyde with 3% sucrose in ultrapure water. Samples were dehydrated using an ethanol series, beginning at 30% ethanol and ending at 100%. Samples were then critically point dried and coated with osmium prior to imaging (LEO Gemini 1525). Rheological characterization of hydrogels was performed following a recommended protocol for hydrogels for tissue engineering applications^70^. Testing was performed on an Anton-Paar MCR 302 rheometer with a 25-mm parallel-plate fixture under strain-controlled conditions. Before sample loading, the lower Peltier cell was set to 4 °C. After sample loading and lowering of the measuring system, mineral oil was applied to the edges of the fixture and the system was enclosed within a solvent trap to prevent sample dehydration. Once the rheological measurement was started, the Peltier cell was rapidly ramped up to 37 °C. All samples were prepared fresh. Time sweeps were performed for 120 min. at 0.1% strain and 10 rad/s. All sweeps were run in triplicate (n = 3).

### Cholangiocyte Encapsulation and Culture

Immortalized mouse small cholangiocytes (SV40SM) were expanded as previously described^55^. All cell culture materials were obtained from Gibco unless otherwise specified. Cells were expanded in MEM supplemented with 10% FBS, L-glutamine, and penicillin/streptomycin. Prior to passaging, dECM pre-gel was formulated using 10 mg/mL dECM digests buffered with 10x PBS, pH adjusted with 1 M NaOH (Sigma) to pH = 7.4, and diluted with ultrapure H_2_O to 5 mg/mL^40^. All solutions and pre-gel were kept chilled or on ice during gel formation. Cells were then passaged using TrypLE Express and re-suspended in a small volume of media, which was then mixed with the 5 mg/mL pre-gel to a final cell concentration of 2 × 10^5^cells/mL gel, or at 1.5 × 10^6^cells/mL and 5 × 10^4^cells/mL for multi-color concentration studies. The cells suspended within the pre-gel were cast into coverslip-bottomed culture dishes and immediately placed within a humidified incubator at 37 °C and 5% CO_2_ and allowed to gel for 1 hour. Growth medium was then added on top of gels and changed after 24 hours to William’s Medium E (without phenol red) supplemented with 0.5% FBS, penicillin/streptomycin, 100 nM insulin (Sigma), and 5 μM hydrocortisone (Sigma) as described in^29^. Encapsulation of SV40SM cells within type 1 porcine collagen (Advanced Biomatrix) followed an identical procedure. Cells were also encapsulated within undiluted Matrigel (Corning) pre-gel. Media was changed every other day. Immortalized mouse large cholangiocytes (SV40LG) were also encapsulated in liver dECM gel at 2 × 10^5^cells/mL gel using the same protocol. For SV40SM cultured in monolayer on dECM gels, dECM was gelled as described without cells, followed by addition of a cell suspension on top of the gel. For cultures with varying dECM concentrations, dECM was either diluted with cold PBS or concentrated by lyophilizing and re-suspending to achieve a variety of concentrations.

### Notch Measurement and Inhibition

Samples of liver dECM alone, 2D cultured SV40SM, and dECM-encapsulated SV40SM were assayed for mouse Jagged1 and Notch1 proteins via ELISA according to manufacturer specifications (LifeSpan Biosciences catalog no. LS-F7394 and LS-F14664-1 respectively). Briefly, samples were harvested after 1 and 7 days of culture and frozen at −80 °C until homogenization on the day of assay. Samples were read on a Cytation 3 Plate Reader (BioTek). Values were normalized to cell number per culture. For Notch inhibition experiments, cultures of encapsulated eGFP-SV40SM were incubated with culture media supplemented with 25 µM of the γ-secretase inhibitor L-685,458 (Tocris) and imaged every 48 hours.

### Proliferation Measurements

Proliferation of SV40SM and SV40LG was evaluated by encapsulating equal amounts (2 × 10^5^cells/mL gel) within dECM at volumes of 250 µL. Samples (n = 3) were harvested every 48 hours for 7 days and frozen at −20 °C until assayed. An equivalent number (50,000) of cells were plated on to wells within a 6-well culture plate to compare proliferation on traditional tissue culture plastic. 2D samples (n = 3) were trypsinized every 48 hours as described above and frozen at −20 °C. Double-stranded DNA content was then measured using PicoGreen dsDNA Assay Kit as described above. Quantified DNA content was then normalized to an aliquot of manually counted cells to obtain an amount of DNA per cell to present data as number of cells/culture.

### Fluorescent Protein Labeling

SV40SM cells were stably transfected to express either mCherry (red), eGFP (green), or Azurite (blue). Bacterial stabs of *E. coli* expressing specific plasmids were obtained as gifts from Pantelis Tsoulfas and Didier Trono via Addgene: pLV-mCherry (Plasmid 36084), pLV-eGFP (Plasmid 36083), pLV-Azurite (Plasmid 36086), pRSV-Rev (Plasmid 12253), pMDLg/pRRE (Plasmid 12251), pMD2.G (Plasmid 12259)^71,72^. Cultures were expanded overnight in LB broth, with cultures expressing pLV strains grown in low salt LB. Plasmid DNA was isolated using PureLink HiPure Midiprep Kit (Invitrogen). Three separate culture of HEK293T cells were transfected using standard CaCl_2_ methods to produce lentiviral vectors for either mCherry, eGFP, or Azurite transduction. Three separate cultures of SV40SM were then treated with supernatant containing lentivirus and cultured for 3 days without media change. Cells were then passaged and sorted for fluorescence using a BD FacsAria SORP 5-Laser cell sorter. Cells were recovered from sorting, culture and encapsulation then proceeded as described above. Multi-color experiments incorporated equal proportions of all three colors in gridded coverslip bottom 35 mm petri dishes (MatTek) to facilitate multipoint imaging. All cells tested negative for mycoplasma contamination using a MycoProbe kit (R&D Systems).

### Bile Duct Function

Polarization of multi-cellular cysts (Matrigel cultures) or ducts (liver dECM cultures) was visualized under transmission electron microscope imaging. Samples were processed for TEM and imaged using a FEI Tecnai Spirit G2. A separate culture of mCherry-SV40SM was subjected to 1 μM γ-aminobutyric acid (GABA) for 7 days to induce expression of large cholangiocyte proteins^60^. Apical transport of cholyl-lysyl-fluorescein (CLF) was examined by incubating GABA-mCherry cells in 5 μM CLF dissolved in HBSS (Gibco) for 1 hour, followed by three washed of warm PBS and 30 minutes incubation in culture media. Separate cultures of GABA-treated mCherry-SV40SM was subjected to Rhodamine-123 (R123) dissolved in William’s Medium for 30 minutes to allow for full diffusion through the dECM gel. R123 samples were then washed three times and incubated in Complete William’s Medium for 30 minutes. Samples were imaged using an incubated Nikon A1 Confocal Laser Microscope.

### 3D Bile Duct Imaging

Cultures of SV40SM encapsulated within gels of either type 1 collagen, Matrigel, or liver dECM were imaged for viability using Live/Dead (Molecular Probes) on a Nikon A1 Confocal Laser Microscope. Fluorescently transduced cultures were imaged using an incubator attachment to facilitate multipoint live cell imaging, with media briefly replaced with FluoroBrite DMEM supplemented with ProLong Live Antifade Reagent during imaging. After imaging, media were switched back to the modified William’s Medium E described above. Images were processed for maximum intensity Z projections, blended volume view, or Z-depth coding using NIS Elements (Nikon) software.

### Image Quantification

Image quantification of 3D image z-stacks was performed using the Filament Tracer function of Imaris (Bitplane) image analysis software. Identical parameters, including diameter of branch beginning, seed points, and contrast thresholds were used to analyze images from notch inhibition experiments and varying dECM weight percent experiments. Aggregates of cells were not counted in total counts of duct structures per scan area.

### Immunohistochemistry

Immunostaining was performed as described in^73^ and^74^ with antibodies outlined in Table 1. Briefly, cultures were fixed overnight by immersion in 4% paraformaldehyde at 4 °C and subjected to cryoprotection in 30% sucrose in phosphate-buffered saline (PBS) overnight at 4 °C. Afterwards, the cultures were embedded in tissue-freezing medium (Tissue-Tek, Triangle Biomedical Sciences) and cut into sections (10–12 µm) with a cryostat. Slides were washed with TBST (TBS + 0.05%Tween 20) for 10 min at room temperature (RT) and incubated for 30 min with blocking solution (2% Blocking reagent [Roche, cat.11096176001] + 20% Fetal Bovine Serum in Maleate Buffer). Primary antibodies diluted in blocking solution were added and incubated overnight in a humidified chamber at RT. Afterwards, sections were washed 3 times for 10 minutes with TBST. Next, secondary antibodies diluted in blocking solution were incubated for 2–3 hours at RT. Sections were washed 3 times for 5 minutes with PBS, incubated with 4′,6-diamidino-2-phenylindole (DAPI) for 5 minutes at RT, washed for 5 minutes with PBS, dipped twice in dH2O, and covered with mounting medium (ProLong Gold, Thermo Fisher, cat. P36930).

### Statistical Analysis

Statistical significance was determined using paired, 2-tailed Student’s T-tests.

**Table 1:** Primary and secondary antibodies used in immunostaining experiments.

Antibody	Species	Company	Dilution	Cat. number
**Primary Antibodies**				
E-cadherin	Rat	NOVEX	1:5,000	13–1900
Sox9	Rabbit	Millipore, Cat.	1:2,000	AB5535
Sox9	Goat	R&D Systems	1:50	AF3075
Hnf1beta	Rabbit	ProteinTech	1:1,000	12533-1
Cytokeratin-19	Rat	DSHB, U. of Iowa	1:100	TROMA-III
Laminin	Rabbit	Sigma	1:500	L-9393
ZO-1	Rabbit	Thermo Fisher	1:100	40–2200
Claudin-7	Rabbit	Thermo Fisher	1:250	34–9100
**Secondary Antibodies**				
CY3-anti Rat	Donkey	Jackson ImmunoResearch	1:200	712-165-153
CY3-anti Rabbit	Donkey	Jackson Immuno.	1:200	711-165-152
CY3-anti Goat	Donkey	Jackson Immuno.	1:200	705-165-147
Alexa 488-anti Rabbit	Donkey	Jackson Immuno.	1:200	711-545-152
Alexa 488-anti Goat	Donkey	Jackson Immuno.	1:200	705-545-147
Alexa 488-anti Rat	Donkey	Jackson Immuno.	1:200	712-545-153

## Electronic supplementary material

Supplementary Video 1

Supplementary Information
