# Author Correction: Complex bile duct network formation within liver decellularized extracellular matrix hydrogels

**DOI:** 10.1038/s41598-022-26508-0

**Published:** 2023-01-17

**Authors:** Phillip L. Lewis, Jimmy Su, Ming Yan, Fanyin Meng, Shannon S. Glaser, Gianfranco D. Alpini, Richard M. Green, Beatriz Sosa-Pineda, Ramille N. Shah

**Affiliations:** 1 Biomedical Engineering, Northwestern University, Evanston, IL USA; 2 Simpson Querrey Institute, Northwestern University, Chicago, IL USA; 3 Research Central Texas Veterans Health Care System, Temple, TX USA; 4 Baylor Scott & White Health Digestive Disease Research Center, Temple, TX USA; 5 Medical Physiology, Texas A&M University College of Medicine, Temple, TX USA; 6 Division of Gastroenterology and Hepatology, Northwestern University, Chicago, IL USA; 7 Nephrology, Northwestern University, Chicago, IL USA; 8 Materials Science and Engineering, Northwestern University, Evanston, IL USA; 9 Surgery (Transplant Division), Northwestern University, Chicago, IL USA

Correction to: *Scientific Reports* https://doi.org/10.1038/s41598-018-30433-6, published online 15 August 2018

This Article contains an error in Figure 7, where panels C and D are duplicated. The correct Figure 7 and the accompanying legend appear below.

**Figure 7:**
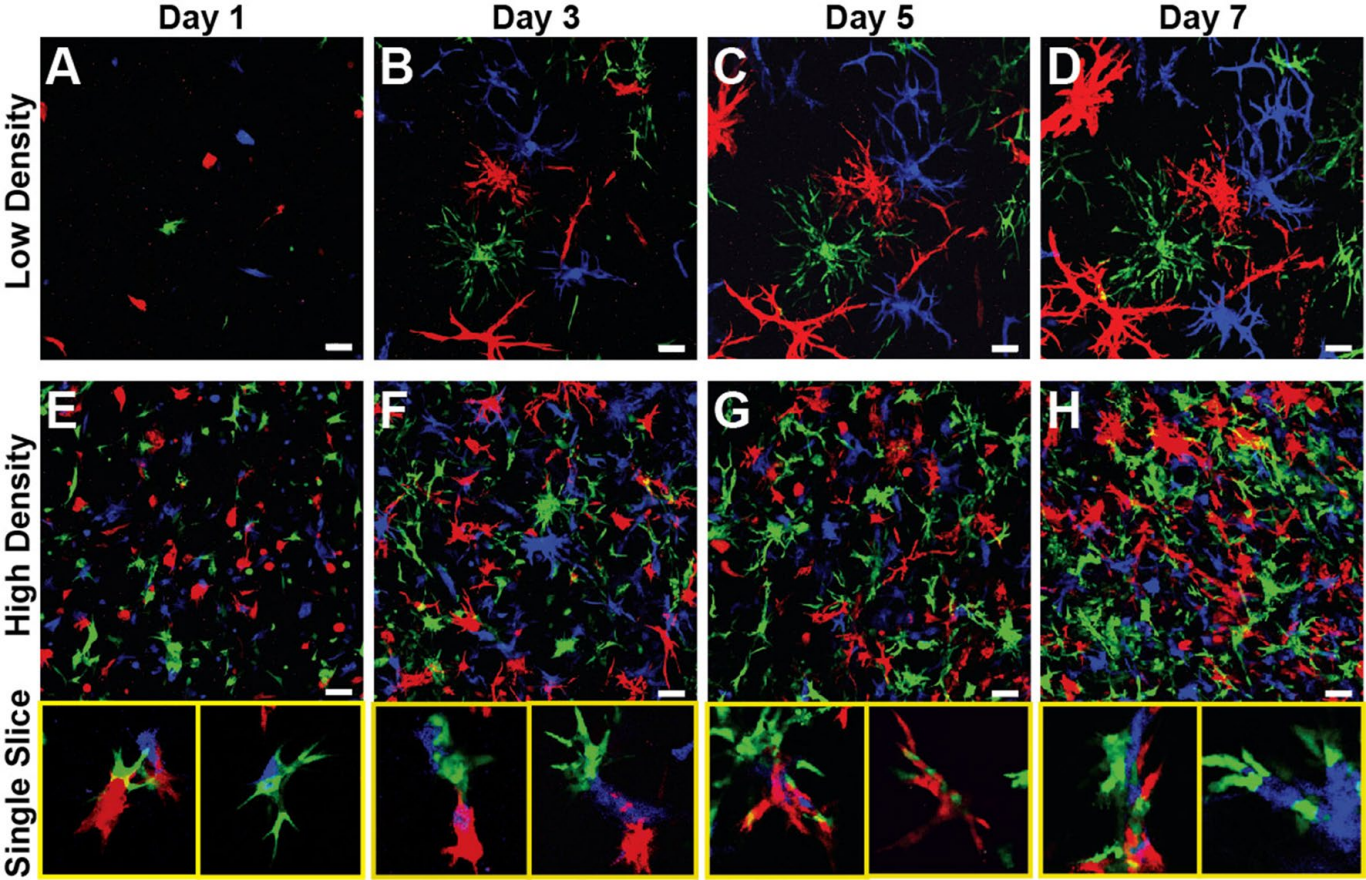
Duct Assembly Model Depends on Proximity. Multipoint imaging of low cell density (5e4 cells/mL) cultures (**A**–**D**) and high cell density (1.5e6 cells/mL) cultures (**E**–**H**). Low density cultures show clonal branching from single cells suspended within the gel, while multi-colored structures are apparent in high density cultures after only a single day in culture because of their close proximity. Representative single z-slices of high density cultures indicate that structures composed of two or three colors are fairly common at all time points. Scale bars = 100 μm.

