# Supplementary material for: Complex bile duct network formation within liver decellularized extracellular matrix hydrogels
Source: Sci Rep. 2018 Aug 15;8:12220. doi: 10.1038/s41598-018-30433-6 (PMC6093899; doi:10.1038/s41598-018-30433-6)
Supplement: Supplementary file 2 — Supplementary Information [file 41598_2018_30433_MOESM2_ESM.docx]

**Complex bile duct network formation within liver decellularized extracellular matrix hydrogels**

Phillip L. Lewis^1,2*^, Jimmy Su^1,2^, Ming Yan^1,2^, Fanyin Meng^3,4^, Shannon S. Glaser^3,4,5^, Gianfranco D. Alpini^3,4,5^, Richard M. Green^6^, Beatriz Sosa-Pineda^7^, Ramille N. Shah^2,8,9^

^1^Biomedical Engineering, Northwestern University;

^2^Simpson Querrey Institute, Northwestern University;

^3^Research, Central Texas Veterans Health Care System;

^4^Baylor Scott & White Health Digestive Disease Research Center;

^5^Medical Physiology, Texas A&M University College of Medicine;

^6^Division of Gastroenterology and Hepatology, Northwestern University;

^7^Nephrology, Northwestern University;

^8^Materials Science and Engineering, Northwestern University;

^9^Surgery (Transplant Division), Northwestern University.

*Corresponding Author: PLL256@u.northwestern.edu

**Supplementary Information**

**Supplementary Figure 1 | Liver dECM Gel Properties are Ideal for Cell Culture.** (**a**) Quantification or residual SDS content before and after 70% ethanol wash. Ethanol reduces SDS content below the detection limit. (b) SEM of liver dECM gel indicates random orientation of fibrous ECM proteins. (**c**) Rheological characterization of liver dECM gel at 37⁰C over time, indicating gelation within minutes and stabilization under 1 hour. Scale bar = 2μm.

**Supplementary Figure 2 | Laminin is Present Throughout Liver dECM and at the Base of Hollow Duct-Like Structures.** Diffuse green (laminin) staining throughout the construct indicates laminin is present in some quantity. Brighter staining is evident at the base of hollow (cyst or duct cross section) structures, indicating mild secretory capacity or concentration of laminin as ducts expand radially. Scale bars = 25μm.


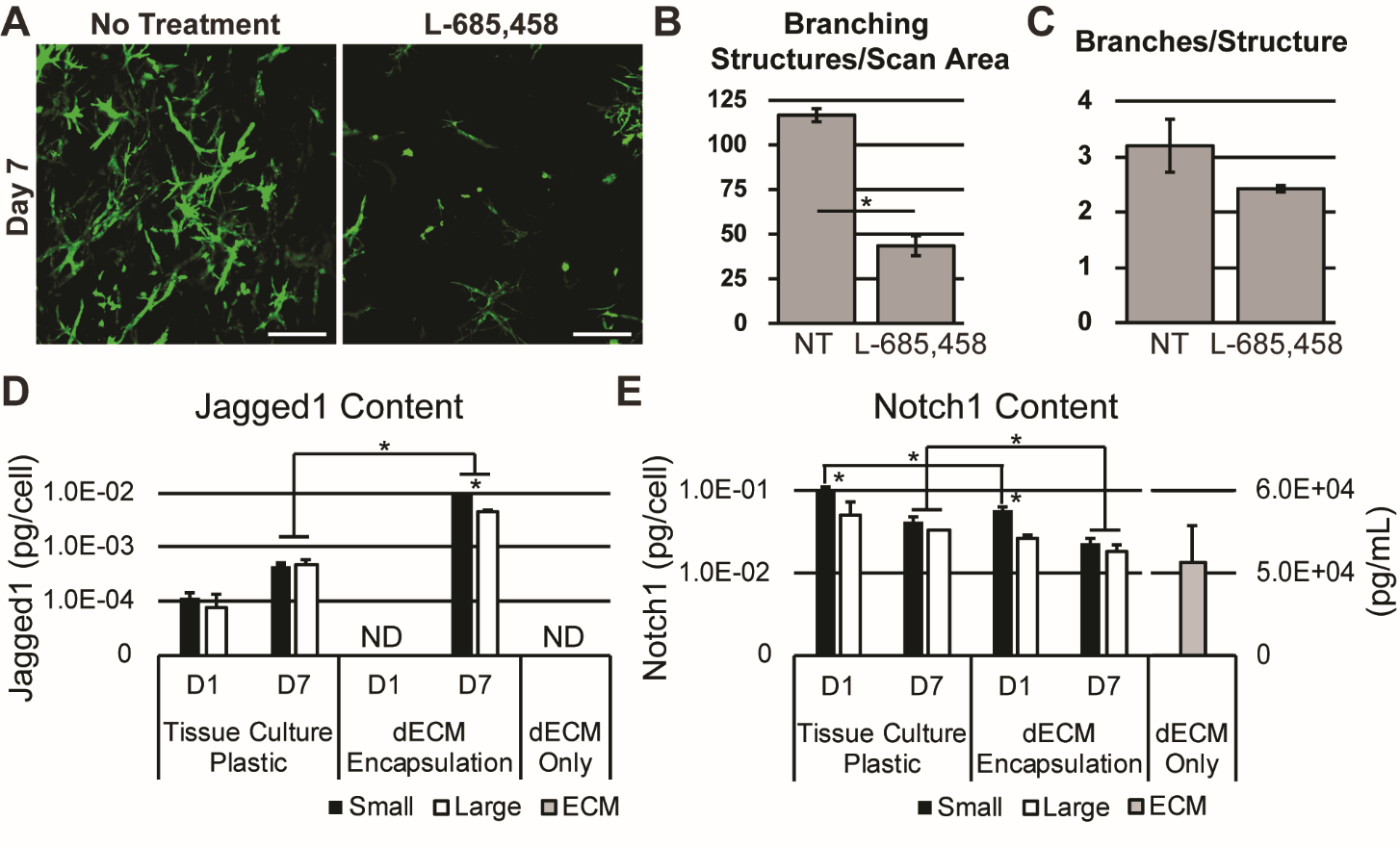


**Supplementary Figure 3 | Notch Inhibition Effects on Branching and Jagged/Notch dECM Content.** (**a**) eGFP-SV40SM either with no treatment (NT) or with 25 µM L-685-458. Images are maximum intensity z-projections. (**b**) Quantified number of branching structures per image. (**c**) Number of branches per structure, indicating no significant decrease after notch inhibition. (**d**) Detection of Jagged1 in SV40SM and SV40LG cultured on standard tissue culture plastic and encapsulated with dECM. Samples are normalized to cellular content. Jagged1 was not detected (ND) in dECM only or in Day 1 culture. (**e**) Notch1 receptor content within cultures. dECM contains large amounts of Notch1 leftover from decellularization. Error bars ±SD, n=3, *=p<0.05, scale bars = 250µm.

**Supplemental Figure 4 | Gel Thickness and Culture Time Influences Branching Structure.** (**a-c**) Z-depth coded images of maximum intensity projections shown in **Figure 2 d-f**. A homogenous distribution of cells throughout the gel influences the resulting structure. (**d**) A thin gel can lead to branching in a confined space when cultured over longer periods of time (21 days). The majority of branches in this image are within the 300-200 μm depth range.

**Supplementary Figure 5 | Cholangiocyte Seeding Strategy Influences Structure Formation.** (**a-b**) Schematic of top-seeding cholangiocytes on a pre-formed gel, and the resulting bud into the dECM gel. (**c**) Transmitted light image of a bud formed from top-seeding. Arrows indicate preliminary branching. (**d**). Z-depth coded image of (**c**), initially visualized using live/dead. (**e**) Confocal cross section of a bud structure, indicating incomplete closure. Scale bars = 250μm


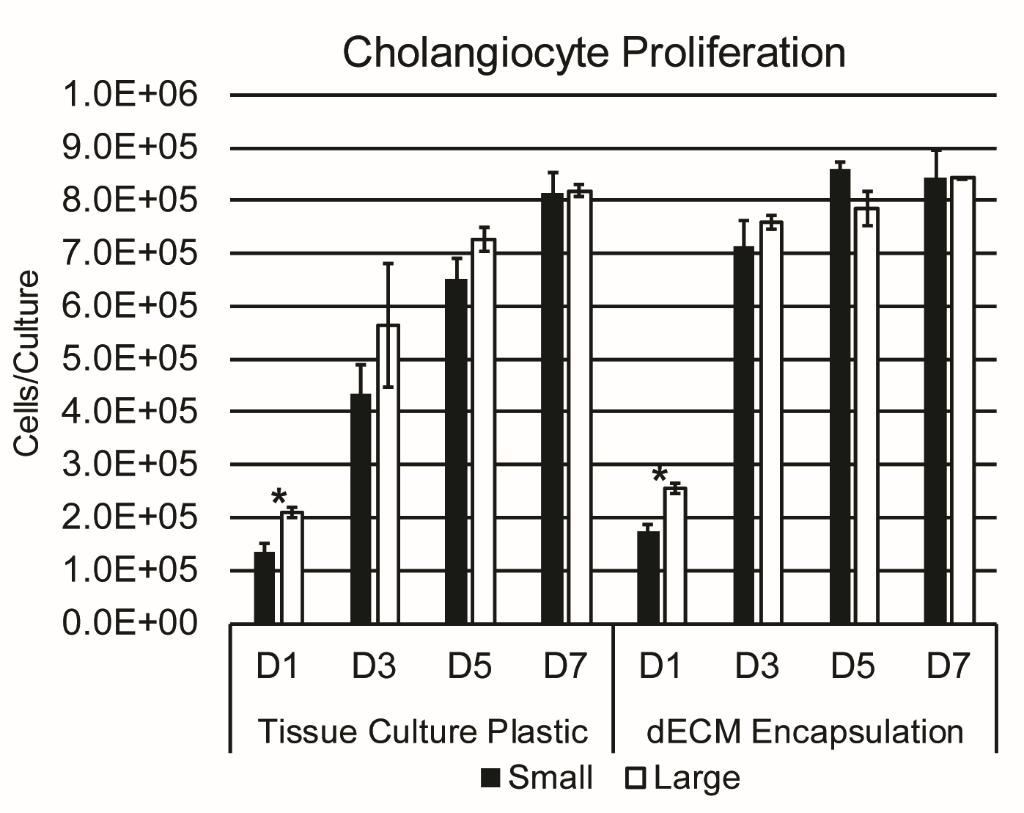


**Supplementary Figure 6 | Small and Large Cholangiocytes Proliferate Identically.** Comparison of SV40SM and SV40LG grown in 2D on standard tissue culture plastic (35mm dish, 6-well plates) and encapsulated within liver dECM. There is no significant difference after 7 days in either culture condition. Error bars ± SD, n=3, *=p<0.05.


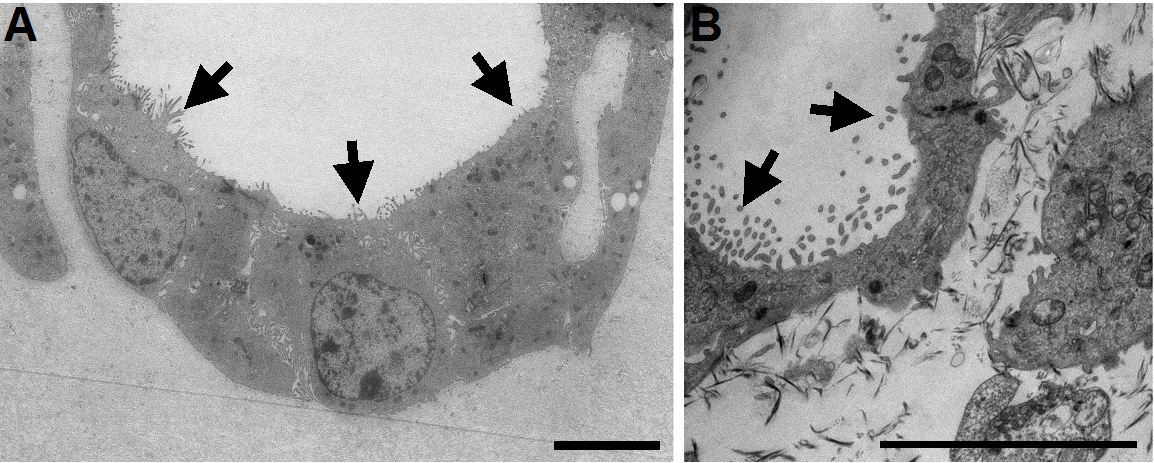


**Supplementary Figure 7 | Cysts within Matrigel and Liver dECM Show Polarization.** TEM image of cholangiocytes cultured within Matrigel (**a**) and liver dECM (**b**) after 7 days. Arrows indicate the apical surface of cysts/ducts as evident by microvilli.


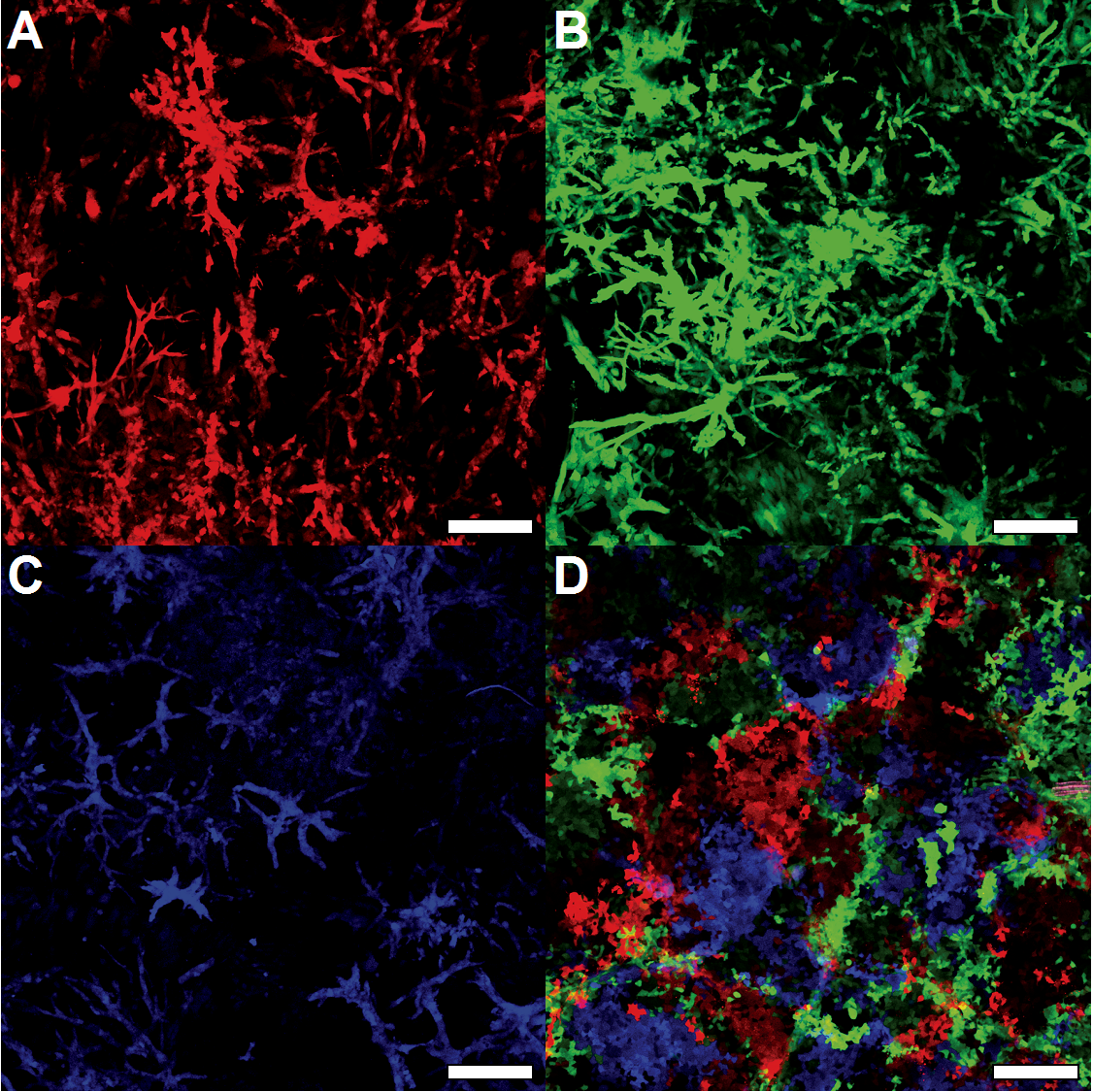


**Supplementary Figure 8 | Cholangiocytes of Different Colors Behave Identically.** (**a-c**) Cholangiocytes expressing mCherry (red, **a**), eGFP (green, **b**), or Azurite (blue, **c**) all form ducts when encapsulated within liver dECM, indicating fluorescent protein transfection does not influence this aspect of their biology. (**d**) All 3 cell lines cultured in 2D form a homogeneous mosaic of equal colors.

**Supplementary Figure 9 | Formation of Multi-Colored Structures is Dependent on Proximity.** (**a-b**) Examples of additional multi-colored duct-like structures. Asterisks indicate mosaic-like ducts that assembled early during formation, arrows indicate duct structures that came together and merged later in culture. Images are maximum intensity z projections. Scale bars = 200μm
